# Socioeconomic Status and Survival in Sarcomas of the Breast: Nationwide Cohort Study

**DOI:** 10.1245/s10434-026-19688-w

**Published:** 2026-04-24

**Authors:** Carl Sars, Enoch Yi-Tung Chen, Anna L. V. Johansson, Paul W. Dickman, Jan Frisell, Helena Sackey, Ebba K. Lindqvist

**Affiliations:** 1 Department of Molecular Medicine and Surgery, Karolinska Institutet, Stockholm, Sweden; 2 Department of Medical Epidemiology and Biostatistics, Karolinska Institutet, Stockholm, Sweden; 3 Division of Cancer, Department of Breast, Endocrine Tumors and Sarcoma, Karolinska Comprehensive Cancer Center, Karolinska University Hospital, Stockholm, Sweden; 4 Department of Clinical Science and Education, Stockholm South General Hospital, Karolinska Institutet, Stockholm, Sweden; 5 Department of Surgery, Stockholm South General Hospital, Stockholm, Sweden

## Abstract

**Introduction:**

Socioeconomic status (SES) has been associated with survival in breast cancer; yet its role in sarcomas of the breast remains poorly studied. We aimed to investigate whether individual-level SES, approximated by educational attainment and household income, is associated with overall survival in a nationwide cohort of women with sarcomas of the breast within a universal healthcare system.

**Methods:**

We conducted a nationwide cohort study by using linked Swedish population registers. Women diagnosed with an incident borderline or malignant phyllodes tumor (PT), angiosarcoma, or soft-tissue sarcoma of the breast from 1993 to 2018 were included and followed through 2019. Socioeconomic status was defined by highest attained education and household disposable income. The primary outcome was all-cause death. Hazard ratios (HRs) with 95% confidence intervals (CIs) were estimated by using multivariable Cox proportional hazards regression.

**Results:**

A total of 473 women were included (median age, 51 years). The cohort comprised 198 borderline PT, 179 malignant PT, 25 angiosarcomas, and 71 soft-tissue sarcomas. Median follow-up was 13.1 years. In multivariable analyses, educational level was not associated with overall survival (OS) (≤9 vs. >13 years, HR 1.29, 95% confidence interval [CI] 0.76–a2.22; 10–13 vs. >13 years, HR 1.16, 95% CI, 0.71–1.89]). Lower household income was associated with worse OS (Q1 vs. Q5, HR 2.35, 95% CI 1.22–4.52; Q2 vs. Q5, HR 1.98, 95% CI 1.06–3.7).

**Conclusions:**

Lower income, but not education level, was associated with worse OS in patients with sarcomas of the breast. These results underscore the presence of survival disparities among patients with rare breast tumors, even within a universal healthcare system.

**Supplementary Information:**

The online version contains supplementary material available at 10.1245/s10434-026-19688-w.

Sarcomas of the breast are rare neoplasms that encompass a heterogeneous group of mesenchymal and fibroepithelial tumors, posing significant diagnostic and therapeutic challenges.^1–3^ Collectively, these entities account for approximately 1% of all soft-tissue tumors in the breast.^4^ Angiosarcoma is the most common category of mesenchymal breast tumors and may occur *de novo* or secondary to prior radiation therapy or lymphedema.^5^ Phyllodes tumors (PT), a prominent category of fibroepithelial tumors, are graded as benign, borderline, or malignant. Malignant PTs may undergo sarcomatous transformation or coexist with other sarcomas.^2,6^ The overlapping biological and clinical features of these rare neoplasms often complicate their identification and classification.^7,8^ Surgery is the main treatment and factors influencing survival remain poorly understood.^9^

Socioeconomic status (SES), typically assessed through factors, such as educational level or income, has been linked to differences in cancer incidence, progression and outcomes, breast cancer included.^10–12^ In settings with publicly funded health care systems, disparities in outcomes may reflect not unequal access to treatment per se, but rather inequalities in the practical ability to access and engage with available care, including factors, such as taking time off work or managing childcare.^13–15^

A limited number of studies have evaluated the association between SES and outcome in sarcomas of the breast. Published data are mainly confined to cohort studies using census tract-level neighborhood SES indices, insurance coverage, or race as proxies for SES, none of which capture individual-level socioeconomic heterogeneity with sufficient precision. One study reported no association between SES and overall survival (OS) in women with malignant PT, although the authors themselves noted that the omission of individual-level SES data was a key methodological limitation. Studies relying on area-level or insurance-based proxies are further limited by selection bias, incomplete registries, and restricted generalizability to healthcare systems outside the United States.^16–18^

The optimal method for monitoring and evaluating the effectiveness of cancer care is through the population-based study of cancer survival, which is only possible using data collected by population-based cancer registries. On the basis of the long history of high-quality data available in Sweden, we sought to investigate the possible association between SES and survival in sarcomas of the breast in a population with general access to a well-established public healthcare system. Crucially, examining SES-related survival disparities within a universal healthcare system provides a unique opportunity to isolate the effect of socioeconomic factors from differential access to care, a confound that is difficult to disentangle in mixed or private insurance-based systems. Our hypothesis was that women of lower individual-level SES, approximated by low education level or household income, have poorer OS compared with those with higher SES.

## Methods

### Design

This nationwide cohort study examined the long-term survival in individuals diagnosed with sarcomas of the breast between January 1, 1993, and December 31, 2018. Patients were followed until the date of death, date of emigration, or end of follow-up (December 31, 2019), whichever occurred first. The data were collected from five nationwide Swedish registries (presented below). The study was approved by the Swedish Ethical Review Authority (diary numbers 2017/641-31/1 and 2010-1950-31/4, amendments 2018/1293-32, 2022-02992-02, and 2023-06429-02). Informed consent was waived as the study was registry-based.^19^ This manuscript followed the Strengthening the Reporting of Observational Studies in Epidemiology (STROBE) reporting guideline.

### Setting and Data Sources

This study utilized comprehensive cross-linkage among Swedish national registries, enabled by the unique personal identification number (PIN) assigned to every Swedish resident.^20^ The PIN allows for accurate individual-level linkage across various health and quality registers. Tumor category, TNM, socioeconomic information, comorbidity, surgical treatment, and data on dates and causes of death and emigration were obtained through linkage with the following registers. The Swedish Cancer Register (SCR) was established in 1958 and records more than 96% of all malignancies.^21^ From the SCR we identified patients with a first occurrence of a sarcoma of the breast confirmed by histopathological review, diagnosed between 1993 and 2018. Sarcomas of the breast were categorized into four tumor categories: borderline PT, malignant PT, angiosarcoma, and soft-tissue sarcoma, by using the International Classification of Diseases for Oncology 2nd edition (ICD-O-2) morphology codes (eTable 1 in the Supplement). We were unable to distinguish primary from secondary (e.g., radiation-induced) angiosarcomas within the registry data, which represents a limitation, as these entities may carry distinct prognoses. From the SCR we also retrieved date of diagnosis, age at diagnosis and tumor stage (T and N category in SCR are based on either clinical or histopathological stage information, i.e., some patients only have clinical and some only have histopathological staging). Information on date of death was obtained through the Cause of Death Register (CDR), which is virtually complete (>99%).^22^

Data on surgical treatment and comorbidities were retrieved from the National Patient Register (NPR), which includes nationwide coverage of in-patient admissions since 1987 and specialist outpatient visits since 2001, classified using ICD codes with an overall high validity.^23,24^ From the NPR, we obtained data on surgical treatment and comorbidities. In this study, we defined comorbidity (categorized score 0 or ≥1) according to the most well-validated version of the Charlson comorbidity index (CCI), not counting the current breast tumor.^25^ All comorbidity conditions were captured in the time frame from 6 years before until 1 year preceding the diagnosis of the current breast tumor. This time window has been shown to be optimal in order to reach the highest validity while avoiding the inclusion of potentially cancer-related comorbidities.^26^

Socioeconomic information was obtained from the Longitudinal Integrated Database for Health Insurance and Labor Market Studies (LISA), which provides annual sociodemographic data on all residents of Sweden.^27^ We extracted data on household disposable income, highest attained educational level, and marital status for the year preceding the current breast tumor. Migration status and date of emigration were obtained from the Total Population Register.^28^ Information on patient ethnicity was not available for the cohort.

### Study Population

The study population included all women residing in Sweden between 1993 and 2018 and recorded in the Multi-generation Register,^29^ i.e., born 1932 or later. From this population, inclusion criteria were patients, aged 18–70 years, with a first occurrence of a borderline or malignant PT or other sarcoma of the breast on histopathological review, reported to the SCR from 1993 to 2018, with follow-up until 2019. Cases where the date of cancer diagnosis was registered on or after the date of death were excluded, because this likely reflected autopsy findings (i.e., incidental cases with no follow-up).

### Exposures and Definition of Socioeconomic Status

The exposure in this study was individual-level SES based on highest attained level of education up until 1 year prior to cancer diagnosis. We divided the level of highest attained education into three categories: ≤9 years; 10–13 years; or >13 years, corresponding to completion of primary, secondary, or tertiary education in the Swedish system. These categories reflect meaningful and distinct educational thresholds with established clinical and epidemiological relevance, rather than arbitrary quantile-based divisions. For income, quintiles were used in accordance with standard practice in Swedish register-based research, allowing for detection of dose-response patterns across the income distribution. We acknowledge that the resulting subgroup sizes are modest, particularly in tumor-category-specific analyses, which may have limited statistical power; this is addressed in the Limitations. We used education as the primary variable for SES, because previous research suggests an inverse relationship between education and both seeking medical care and overall mortality.^30,31^

In the *a priori* specified sensitivity analysis, we used individual-level household income as SES variable. Income data from the year preceding diagnosis were categorized into calendar year-specific quintiles to account for inflation and minimize reverse causation (e.g., income loss due to illness). We did not fit models containing both income and education to maintain statistical power and to avoid collinearity (see below).

### Outcomes

The main outcome was all-cause death, with OS defined as time from surgery to all-cause death. Hazard ratios (HR) were estimated by using Cox proportional hazards regression.

### Statistical Analysis

The baseline characteristics of the study population were described using frequencies (*n*), percentages (%), and median [interquartile range (IQR)], as appropriate, by the exposure variables. Overall survival was estimated by using the Kaplan-Meier method. Hazard ratios and 95% confidence intervals (CIs) were estimated by using the Cox proportional hazards regression, with the highest SES level as reference in a confounder-adjusted model with time since surgery as the underlying timescale. Patients with missing information on any covariates were excluded (*n* = 8), leaving 465 patients (98.3%) eligible for complete case analysis in the main model. Tests based on scaled Schoenfeld residuals showed no evidence against the proportional hazards assumption.

We used a directed acyclic graph (DAG) for covariate selection in our statistical modelling (eFig. 1). The use of a DAG leveraged our *a priori* subject-matter knowledge about causal structures at the design phase of the study. The DAG helped to identify the most parsimonious set of covariates to be adjusted for. This prevented loss of statistical power and helped to avoid problems, such as collider-stratification bias (adjusting for nonconfounders) and collinearity. Another advantage is that covariate selection via a DAG limits the problems with a traditional stepwise regression procedure, which might increase non-exchangeability. A two-step approach for covariate adjustment was employed. First, we considered four different covariates: age at diagnosis (continuous), comorbidity, tumor category (borderline phyllodes, malignant phyllodes, angiosarcoma, and soft-tissue sarcomas), and surgical treatment (breast conserving or mastectomy) as possible confounders. Second, we created a DAG that rendered a minimally sufficient set for covariate adjustment, leaving only age as the single adjusted variable (confounder) in our model when examining the association between the exposure (SES) and outcome (survival).

In our main model, we adjusted for age only, based on our primary DAG (eFig. 1) to estimate the total effect of SES on survival. As comorbidity may be considered to lie on the causal pathway between SES and survival, we conducted a secondary analysis with additional adjustment for comorbidity (CCI ≥1 vs. 0), estimating the direct effect of SES not mediated by comorbidity (eFig. 2). This analysis is intended as a complementary model, not a replacement of the main analysis.

### Sensitivity Analysis

Socioeconomic status is multidimensional, and other factors besides education could be associated with survival. We therefore performed a sensitivity analysis to test the findings from the main analysis where education served as the SES variable, in which we instead used household disposable income as an alternative SES variable. Hazard ratios were estimated by using Cox proportional hazards regression with the highest income level as the SES variable as reference category, adjusted for age at diagnosis (continuous) as the sole confounder identified by the DAG. Patients with missing covariate data were excluded from the analysis (*n* = 3), leaving 470 individuals (99.4%) eligible. All analyses were performed in Stata 18 (StataCorp. 2024. Stata Statistical Software: Release 18. College Station, TX: StataCorp LLC).

## Results

### Patient Characteristics and Tumor Category Data

The study cohort included 473 patients with borderline or malignant PT, angiosarcoma, or soft-tissue sarcoma of the breast. These patients were identified after excluding 52 of the 525 initially found who had sarcoma at sites other than the breast. The median (IQR) age was 51 (44–59) years, including 198 patients with borderline PT (50 [42–56] years), 179 with malignant PT (51 [45–59] years), 25 with angiosarcoma (65 [54–65] years), and 71 with soft-tissue sarcomas of the breast (54 [47–61] years). The median follow-up time was 13.1 (95% CI 11.6–14.2) years for the entire cohort, 13.2 years (95% CI 11.5–15.1) for borderline PT, 12.1 years (95% CI 9.2–15.2) for malignant PT, 10.2 years (95% CI 6.4–18.2) for angiosarcoma, and 13.8 years (95% CI 10–17.3) for soft-tissue sarcomas of the breast. Baseline characteristics stratified by education level and by tumor category are presented in eTable 2.

**Fig. 1:**
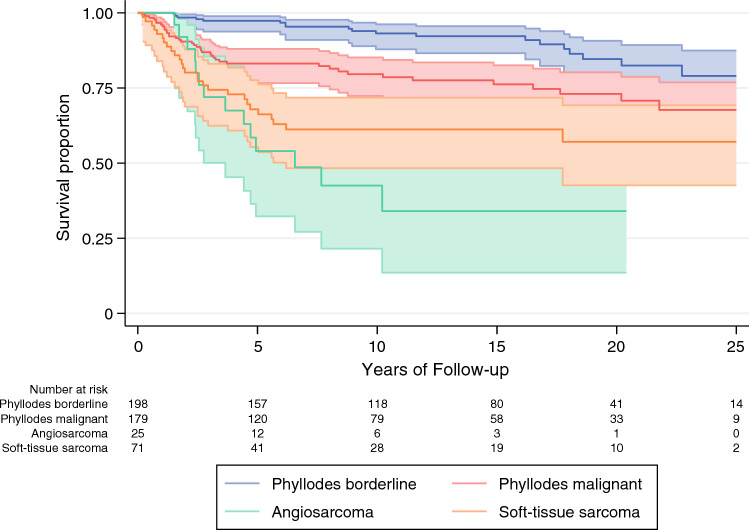
Kaplan-Meier survival estimates by tumor category. The shaded areas indicate 95% confidence intervals for survival estimates. Data source: Swedish Cancer Register, Cause of Death Register, National Patient Register, and LISA database. Study population: Women aged 18–70 years diagnosed with an incident sarcoma of the breast in Sweden, 1993–2018, with follow-up through December 31, 2019

There were 102 deaths during follow-up and the cohort-wide 5-year, and 10-year OS proportions were 85.1% (95% CI 81.5–88.1) and 80.4% (95% CI 76.2–83.9), respectively. Survival estimates for each tumor category are presented in Fig. 1.

### Socioeconomic Status—Educational Attainment

**Fig. 2:**
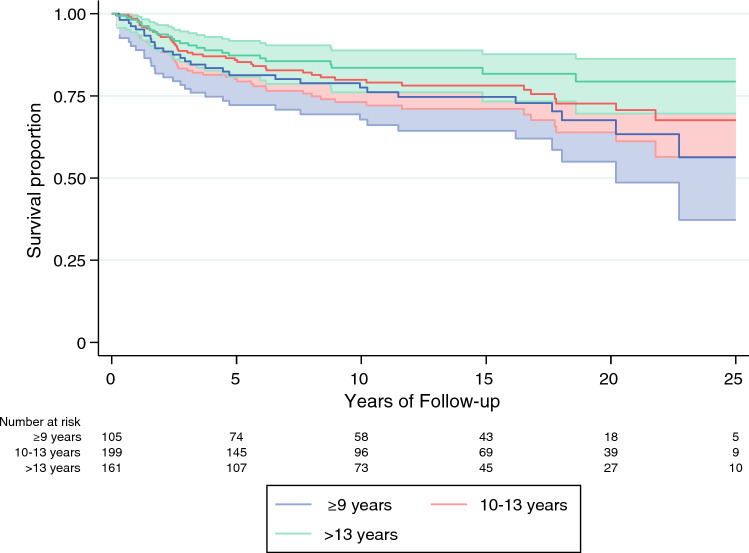
Kaplan-Meier survival estimates by level of highest attained education. The shaded areas indicate 95% confidence intervals for survival estimates. Data source: Swedish Cancer Register, Cause of Death Register, National Patient Register, and LISA database. Study population: Women aged 18–70 years diagnosed with an incident sarcoma of the breast in Sweden, 1993–2018, with follow-up through December 31, 2019

**Fig. 3:**
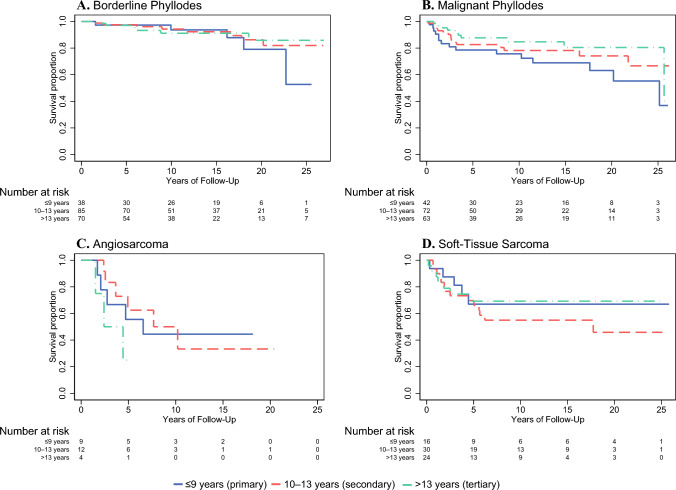
Kaplan-Meier survival estimates by education level for different tumor categories. Data source: Swedish Cancer Register, Cause of Death Register, National Patient Register, and LISA database. Study population: Women aged 18–70 years diagnosed with an incident sarcoma of the breast in Sweden, 1993–2018, with follow-up through December 31, 2019

Baseline characteristics stratified by education level are presented in Table 1. Across education levels, the age distribution, proportions of immigrants, and marital status were similar, and comorbidity rates were less than 15% overall. The distribution of tumor categories was broadly comparable across education levels, although angiosarcoma appeared somewhat more frequent in the lowest education group (8.6% vs. 6% and 2.5% in secondary and tertiary groups, respectively); yet rates of mastectomy were higher among those with a lower education level. Median follow-up was 16.3 years (95% CI 11.8–17.3) for patients with ≤9 years of education, 13.2 years (95% CI 11.1–15.2) for 10–13 years, and 11.1 years (95% CI 9.2–13.1) for >13 years of education and was broadly similar across groups. Kaplan-Meier survival estimates by education level and by education level within each tumor category are presented in Figs. 2 and 3, respectively. The results from the Cox regression analyses are presented in Table 2.

**Table 1:** Patient characteristics by attained education level

	Highest attained education^a^				
	≤9 years (primary)	10-13 years (secondary)	>13 years (tertiary)	Missing	Total
N (%)	105 (22.2)	199 (42.1)	161 (34)	8 (1.7)	473 (100)
*Age at diagnosis*					
18–29	8 (7.6)	11 (5.5)	12 (7.5)	2 (25)	33 (7)
30–39	4 (3.8)	12 (6)	20 (12.4)	3 (37.5)	39 (8.2)
40–49	20 (19.0)	60 (30.2)	58 (36.0)	1 (12.5)	139 (29.4)
50–59	39 (37.1)	66 (33.2)	40 (24.8)	1 (12.5)	146 (30.9)
≥60	34 (32.4)	50 (25.1)	31 (19.3)	1 (12.5)	116 (24.5)
*Tumor category*					
Phyllodes borderline	38 (36.2)	85 (42.7)	70 (43.5)	5 (62.5)	198 (41.9)
Phyllodes malignant	42 (40)	72 (36.2)	63 (39.1)	2 (25)	179 (37.8)
Angiosarcoma	9 (8.6)	12 (6)	4 (2.5)	0 (0)	25 (5.3)
Soft-tissue sarcoma	16 (15.2)	30 (15.1)	24 (14.9)	1 (12.5)	71 (15)
*Charlson comorbidity index*					
0	93 (88.6)	178 (89.4)	145 (90.1)	7 (87.5)	423 (89.4)
≥1	12 (11.4)	21 (10.6)	16 (9.9)	1 (12.5)	50 (10.6)
*Immigration status*					
Swedish	81 (77.1)	163 (81.9)	126 (78.3)	0 (0)	370 (78.2)
Non-Swedish	24 (22.9)	36 (18.1)	35 (21.7)	8 (100.0)	103 (21.8)
*Marital status*					
Unmarried	24 (22.9)	50 (25.3)	45 (28)	1 (16.7)	120 (25.5)
Married	60 (57.1)	113 (57.1)	87 (54)	5 (83.3)	265 (56.4)
Registered partnership	0 (0)	0 (0)	1 (0.6)	0 (0)	1 (0.2)
Divorced	18 (17.1)	30 (15.2)	24 (14.9)	0 (0)	72 (15.3)
Widow	3 (2.9)	5 (2.5)	4 (2.5)	0 (0)	12 (2.6)
*Household disposable income quintiles*					
First (Lowest)	19 (18.1)	29 (14.6)	21 (13.0)	3 (37.5)	72 (15.2)
Second	28 (26.7)	35 (17.6)	22 (13.7)	0 (0)	85 (18)
Third	24 (22.9)	48 (24.1)	23 (14.3)	1 (12.5)	96 (20.3)
Fourth	25 (23.8)	50 (25.1)	34 (21.1)	0 (0)	109 (23)
Fifth (Highest)	9 (8.6)	36 (18.1)	61 (37.9)	2 (25)	108 (22.8)
Missing	0 (0)	1 (0.5)	0 (0)	2 (25)	3 (0.6)
*T category*					
Tx	77 (73.3)	144 (72.4)	109 (67.7)	5 (62.5)	335 (70.8)
T1	8 (7.6)	21 (10.6)	15 (9.3)	3 (37.5)	47 (9.9)
T2	11 (10.5)	21 (10.6)	23 (14.3)	0 (0)	55 (11.6)
T3	7 (6.7)	11 (5.5)	9 (5.6)	0 (0)	27 (5.7)
T4	2 (1.9)	2 (1)	5 (3.1)	0 (0)	9 (1.9)
*Lymph node status*					
Nx	78 (74.3)	147 (73.9)	112 (69.6)	5 (62.5)	342 (72.3)
N0	24 (22.9)	49 (24.6)	46 (28.6)	3 (37.5)	122 (25.8)
N1	3 (2.9)	3 (1.5)	3 (1.9)	0 (0)	9 (1.9)
*Distant metastasis*					
Mx	74 (70.5)	144 (72.4)	105 (65.2)	5 (62.5)	328 (69.3)
M0	29 (27.6)	55 (27.6)	54 (33.5)	3 (37.5)	141 (29.8)
M1	2 (1.9)	0 (0)	2 (1.2)	0 (0)	4 (0.8)
*Surgical treatment*					
Breast conserving surgery	66 (62.9)	120 (60.3)	111 (68.9)	8 (100)	305 (64.5)
Mastectomy	39 (37.1)	79 (39.7)	50 (31.1)	0 (0)	168 (35.5)
*Time period*					
1993–1999	22 (21)	53 (26.6)	33 (20.5)	2 (25.0)	110 (23.3)
2000–2009	52 (49.5)	68 (34.2)	54 (33.5)	3 (37.5)	177 (37.4)
2010–2018	31 (29.5)	78 (39.2)	74 (46)	3 (37.5)	186 (39.3)

^a^All percentages represent column proportions within each education level subgroup

*Data source* Swedish Cancer Register, Cause of Death Register, National Patient Register, and LISA database. Study population: Women aged 18–70 years diagnosed with incident sarcoma of the breast in Sweden, 1993–2018, with follow-up through December 31, 2019

**Table 2:** Hazard ratios of OS survival probability by highest attained education, crude and adjusted for age at diagnosis (continuous variable) as the sole confounder, as determined by directed acyclic graph analysis (eFig. 1)

Highest attained education level	Crude HR (95% CI)	Age adjusted HR (95% CI)
*Total cohort*		
>13 years (ref)	1.00	1.00
10–13 years	1.35 (0.83-2.2)	1.16 (0.71-1.89)
≤9 years	1.74 (1.03-2.95)	1.29 (0.76-2.22)
*Borderline phyllodes*		
>13 years (ref)	1.00	1.00
10–13 years	0.99 (0.34-2.86)	0.80 (0.28-2.32)
≤9 years	1.49 (0.45-4.91)	0.77 (0.22-2.59)
*Malignant phyllodes*		
>13 years (ref)	1.00	1.00
10–13 years	1.41 (0.64-3.11)	1.35 (0.61-2.97)
≤9 years	2.16 (0.97-4.82)	1.82 (0.80-4.1)
*Angiosarcoma*		
>13 years (ref)	1.00	1.00
10–13 years	0.33 (0.08-1.41)	0.35 (0.08-1.53)
≤9 years	0.39 (0.09-1.71)	0.55 (0.11-2.79)
*Soft-tissue sarcoma*		
>13 years (ref)	1.00	1.00
10-13 years	1.48 (0.6-3.66)	1.27 (0.51-3.18)
≤9 years	0.96 (0.3-3.04)	0.79 (0.24-2.54)

*OS* overall survival; *HR* hazard ratio; *CI* confidence interval

*Data source* Swedish Cancer Register, Cause of Death Register, National Patient Register, and LISA database. Study population: Women aged 18–70 years diagnosed with incident sarcoma of the breast in Sweden, 1993–2018, with follow-up through December 31, 2019

The confounder-adjusted model showed no statistically significant difference between OS for different educational levels compared with the highest attained education (≤9 years, HR 1.29, 95% CI 0.76–2.22, and 10-13 years, HR 1.16, 95% CI 0.71–1.89).

In the secondary analysis adjusting for comorbidity, the associations between educational level and OS remained directionally similar to the main model. Compared with individuals with tertiary education (>13 years), those with primary education (≤9 years) had an HR of 1.41 (95% CI 0.83–2.4), and those with secondary education (10–13 years) had an HR of 1.2 (95% CI 0.74–1.95). Comorbidity (CCI ≥1 vs. 0) was strongly associated with poorer survival (HR 3.16, 95% CI 1.97–5.07, *p* < 0.001). Adjustment for comorbidity and age at diagnosis did not change the observed association between educational level and OS, suggesting that comorbidity, while prognostically important, may not fully mediate the effect of SES.

Prespecified subgroup analyses within each of the four tumor categories as defined in the *Methods* revealed no statistically significant associations between educational level and survival in crude or age-adjusted models.

### Socioeconomic Status—Household Income

The baseline characteristics of patients categorized by income quintiles are presented in eTable 3. Using inflation-adjusted income by quintiles as SES variable, the confounder-adjusted model showed a statistically significantly higher risk of mortality when comparing the fifth (highest) quintile with the first (lowest) (adjusted HR 2.35, 95% CI 1.22–4.52) and second quintile (adjusted HR 1.98, 95% CI 1.06–3.7) but no statistically significant difference compared with the third or fourth quintile (Table 3).

**Table 3:** Hazard ratios (HRs) of overall survival by household income quintile: crude, age-adjusted, and age + CCI-adjusted models

Household disposable income quintile	Crude HR (95% CI)	Age Adjusted HR (95% CI)	Age + CCI Adjusted HR (95% CI)
*Total*			
Fifth (ref)	1.00	1.00	1.00
Fourth	0.94 (0.46-1.9)	0.79 (0.39-1.59)	0.83 (0.41–1.69)
Third	1.70 (0.9-3.21)	1.30 (0.68-2.48)	1.26 (0.66–2.4)
Second	2.39 (1.28-4.43)	1.98 (1.06-3.7)	1.99 (1.07–3.71)
First	2.45 (1.28-4.70)	2.35 (1.22-4.52)	1.94 (1–3.77)
*Borderline phyllodes*			
Highest (ref)	1.00	1.00	1.00
Fourth	1.27 (0.18–9.03)	0.86 (0.12–6.17)	0.93 (0.13–6.66)
Third	1.61 (0.23–11.41)	0.85 (0.12–6.3)	0.99 (0.13–7.33)
Second	4.37 (0.88–21.69)	2.89 (0.57–14.56)	3.03 (0.6–15.22)
Lowest	7.19 (1.49–34.73)	8.95 (1.77–45.17)	6.98 (1.33–36.56)
*Malignant phyllodes*			
Highest (ref)	1.00	1.00	1.00
Fourth	1.33 (0.48–3.68)	1.24 (0.45–3.43)	1.14 (0.41–3.16)
Third	2.05 (0.78–5.39)	1.73 (0.65–4.61)	1.54 (0.58–4.09)
Second	1.84 (0.64–5.32)	1.77 (0.61–5.12)	1.60 (0.55–4.67)
Lowest	1.77 (0.54–5.83)	1.89 (0.57–6.22)	1.62 (0.49–5.4)
*Angiosarcoma*			
Highest (ref)	1.00	1.00	1.00
Fourth	0.67 (0.13–3.52)	0.89 (0.17–4.65)	0.87 (0.16–4.6)
Third	0.63 (0.13–3.01)	1.65 (0.22–12.23)	1.65 (0.22–12.68)
Second	0.63 (0.10–3.87)	1.50 (0.18–12.58)	1.30 (0.13–12.73)
Lowest	0.39 (0.06–2.41)	0.85 (0.10–6.86)	0.84 (0.10–6.89)
*Soft-tissue sarcoma*			
Highest (ref)	1.00	1.00	1.00
Fourth	~0 (0–∞)	~0 (0–∞)	6.35e-21
Third	0.98 (0.26–3.65)	0.79 (0.21–3.02)	0.80 (0.21–3.04)
Second	3.22 (1.10–9.38)	2.75 (0.93–8.11)	2.72 (0.92–8.03)
Lowest	2.84 (0.89–9.02)	2.48 (0.76–8.06)	2.49 (0.77–8.1)

*OS* overall survival; *HR* hazard ratio; *CI* confidence interval; *CCI* Charlson Comorbidity Index

Models estimated using Cox proportional hazards regression. Reference category: highest (fifth) income quintile. “Age-adjusted” models include age at diagnosis (continuous) ) as the sole confounder, as determined by directed acyclic graph analysis (eFig. 1). “Age + CCI-adjusted” models additionally adjust for Charlson Comorbidity Index (CCI ≥1 vs. 0)

Data source: Swedish Cancer Register, Cause of Death Register, National Patient Register, and LISA database. Study population: Women aged 18–70 years diagnosed with incident sarcoma of the breast in Sweden, 1993–2018, with follow-up through December 31, 2019

**Fig. 4:**
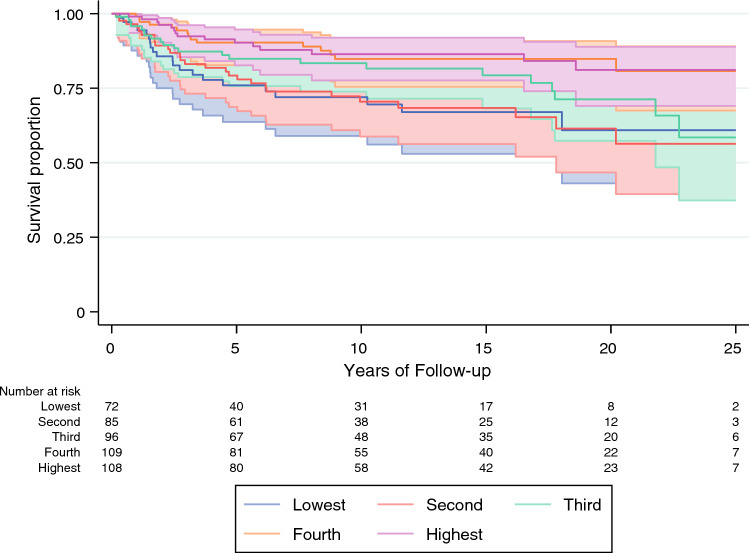
Kaplan-Meier survival estimates by income quintiles. The shaded areas indicate 95% confidence intervals for survival estimates. Data source: Swedish Cancer Register, Cause of Death Register, National Patient Register, and LISA database. Study population: Women aged 18–70 years diagnosed with incident sarcoma of the breast in Sweden, 1993–2018, with follow-up through December 31, 2019

Prespecified subanalysis of different tumor categories with income as SES variable was performed. When stratified by tumor category, low income was particularly associated with lower OS in patients with borderline PT (adjusted HR 8.95, 95% CI 1.77–45.17). For other tumor categories, there were similar trends in point-estimates but not with statistical significance. Kaplan-Meier survival estimates by income quintiles are presented in Fig. 4 and by income quintiles for different tumor categories in the Supplement (eFig. 3).

## Discussion

The findings of this study suggest that among women diagnosed with sarcomas of the breast, those with the lowest income levels had worse survival compared with those with the highest. While lower income was associated with worse overall survival, educational level was not significantly associated with survival in our cohort. This latter finding does not support the hypothesis that education, as an indicator of SES, is associated with OS in this setting. The observed association between lower income level and poorer survival was primarily driven by patients with borderline PT; however, across all tumor categories, point estimates consistently indicated worse OS among women with lower income levels compared with those with the highest.

In both the main model (adjusted for age) and the secondary model (additionally adjusted for comorbidity), HRs for lower education levels suggested worse survival but were not statistically significant. Nevertheless, the consistent direction and magnitude of effect across all models suggest a potential association that warrants further investigation in larger cohorts.

This suggests that comorbidity may not fully mediate the association between education and survival. The attenuation of the effect was modest, indicating that other pathways, beyond comorbidity, may contribute to SES-related survival differences. However, because comorbidity was treated as a mediator rather than a primary exposure, this association should be interpreted with caution.

In this cohort, women who were unmarried or foreign-born, more common in the lowest income quintile, may have less social support, which has been associated with delayed care-seeking and worse cancer outcomes in prior research. Nevertheless, no substantial differences were observed in the distribution of T-category at diagnosis or the proportion of breast-conserving surgery between income groups. SES is a multidimensional construct, and income and education capture different aspects of it. Education is often interpreted as a proxy for health literacy, health-related behaviors, and engagement with health care, whereas income reflects material resources and the ability to manage the practical and financial consequences of illness.^32,33^ Although correlated, these dimensions are not interchangeable. In our study, we therefore examined income and education in separate models to explore their individual associations with survival. Given the relatively small sample size and the potential collinearity between the two measures, we chose not to adjust for both simultaneously. Nonetheless, the observation that only income, but not education, was associated with survival may suggest that financial resources, rather than educational attainment, play a more prominent role in shaping outcomes for patients with sarcomas of the breast.

Understanding the underlying mechanisms driving these disparities is crucial for addressing inequalities and identifying potential areas to improve health care for all patients. The nationwide Swedish Cancer Register enables the study of survival outcomes for all cancer patients, providing a more comprehensive picture of the development of cancer care trends in the population. Population-based survival estimates serve as important indicators of how well the healthcare system diagnoses and treats cancer. This is of interest to patients, clinicians, and healthcare planners alike. Sweden offers universal health care to all its residents. Nevertheless, income level may influence patients’ capacity to spend money on medication and healthcare visits. Similarly, low educational attainment may be associated with lower rates of participation in screening programs, possibly due to lower health literacy or awareness, even when formal access is equal.^34,35^ Borderline PT is notoriously difficult to distinguish from malignant PT, which may impact treatment options (extent of surgery) and follow-up. The findings of our study suggests that the survival difference seen among SES groups may be a sign of healthcare disparities, even in a society that provides universal coverage. Part of the problem might be due to clinician implicit bias, whereby unconscious attitudes or assumptions influence clinical decision-making and patient management.^36^

The survival probability for borderline and malignant PT have previously been approximated to 60–80% at 5 years,^37,38^ and in more recent years to approximately 88% for borderline and 71% for malignant PT.^39^ This is congruent with our results. The long-term survival probability for angiosarcoma was recently estimated at 35–50%,^40^ which our report also confirms. Previously published studies have reported an increased mortality among European women diagnosed with breast cancer and lower SES.^12^ However, studies investigating the impact of SES on survival and other long-term outcomes among women with PT or sarcomas of the breast are scarce. One study examined the impact of crude *neighborhood socioeconomic status* on the mortality rate of PT, where the authors concluded that the mortality was mainly driven by tumor and clinical factors rather than SES.^18^ Two additional studies examined the impact of race. These found differences in tumor characteristics across racial groups but reached conflicting conclusions regarding race-related survival differences.^16,41^ Finally, insurance status has been shown to be associated with worse prognosis, partly mediated by higher tumor stage at presentation.^17^ Although mostly large in sample sizes, these studies are at high risk of selection bias owing to incomplete registries or confined to a single-center. Furthermore, they may not be applicable to other settings or countries.

In summary, while many publications have examined the outcomes of patients with sarcomas of the breast, ours is the first nationwide study evaluating individual-level SES as a risk factor for worse long-term survival outcomes. Furthermore, the divergent findings whereby income but not education was significantly associated with survival represent a nuanced result suggesting that material resources, rather than health literacy alone, may be the more important driver of outcomes in this setting. This distinction has direct implications for how policy interventions should be targeted to reduce health inequities in this patient group.

### Strengths and Limitations

Strengths of this study include its population-based setting, providing a near-complete sample with reliable follow-up in a universal health care setting. The high validity and completeness of the register data used, and the complete follow-up of all participants are among the methodological advantages of the present study. To the best of our knowledge, this is the first report that uniquely incorporates robust, individual-level socioeconomic variables in survival analysis of sarcomas of the breast. Limitations include the possibility of residual confounding from unmeasured or unknown variables, such as health literacy, alcohol consumption, smoking status, or body mass index. However, using a DAG for a causal approach of analysis provides a thorough analysis of potential confounders and minimizes the risk of overfitting models and adjusting for nonconfounders. Disease-specific survival was not reported as an endpoint, as accurate attribution of cause of death for rare tumor entities in registry data is prone to misclassification; all-cause mortality therefore was chosen as the primary endpoint to avoid this potential bias. Future studies incorporating clinical case-by-case review are encouraged to report disease-specific survival alongside overall survival. Furthermore, we could not differentiate primary from secondary angiosarcomas within the registry data, because this information is not systematically captured in the SCR; given their potentially distinct biological behavior and prognosis, this may have introduced heterogeneity within this tumor category.

Owing to the rarity of sarcomas of the breast, chance is a threat to any study of these tumors. The number of income and education subgroups, while methodologically justified, resulted in small cell sizes particularly in tumor-specific stratified analyses, which should be considered when interpreting those results. While our cohort is sizable in the context of previous breast sarcoma research, it is modest compared with cohorts typically seen in studies of breast cancer.

## Conclusion

In this nationwide, population-based cohort study, we found that lower individual-level SES, as approximated by income, but not education level, was associated with worse OS among patients with sarcomas of the breast. These findings suggest that existing health care structures have not addressed the unequal mortality among socioeconomic groups for these rare breast lesions. This implication is particularly concerning for borderline PT, as limited access to surgical care in some settings may be associated with poorer outcomes and could perpetuate health disparities if unaddressed. Therefore, policy interventions are needed to reduce these health inequities and to mitigate the underlying factors contributing to unequal mortality risk among different socioeconomic groups.

## Supplementary Information

Below is the link to the electronic supplementary material.

Supplementary file1 (DOCX 361 KB)

Supplementary file2 (DOCX 34 KB)
