# Supplementary material for: Socioeconomic Status and Survival in Sarcomas of the Breast: Nationwide Cohort Study
Source: Ann Surg Oncol. 2026 Apr 24;33(8):7181–92. doi: 10.1245/s10434-026-19688-w (PMC13337858; doi:10.1245/s10434-026-19688-w)
Supplement: Supplementary file 1 — Supplementary file1 (DOCX 361 KB) [file 10434_2026_19688_MOESM1_ESM.docx]

**Supplemental online content**

Sars C, Chen EY-T, Johansson ALV, et al. Socioeconomic Status and Survival in Sarcomas of the Breast.

**eFigure 1.** DAG model. Correctly adjusted Directed Acyclic Graph (DAG), displaying possible causal associations to build a statistical model

**eFigure 2.** Alternate DAG model. Correctly adjusted Directed Acyclic Graph (DAG), displaying possible causal associations to build a statistical model

**eTable 1.** Morphologic diagnosis according to ICD-O/2

**eTable 2.** Patient characteristics by tumor category

**eTable 3.** Patient characteristics by income level in quintiles.

**eFigure 3.** Kaplan-Meier survival estimates by income quintiles for different tumor categories


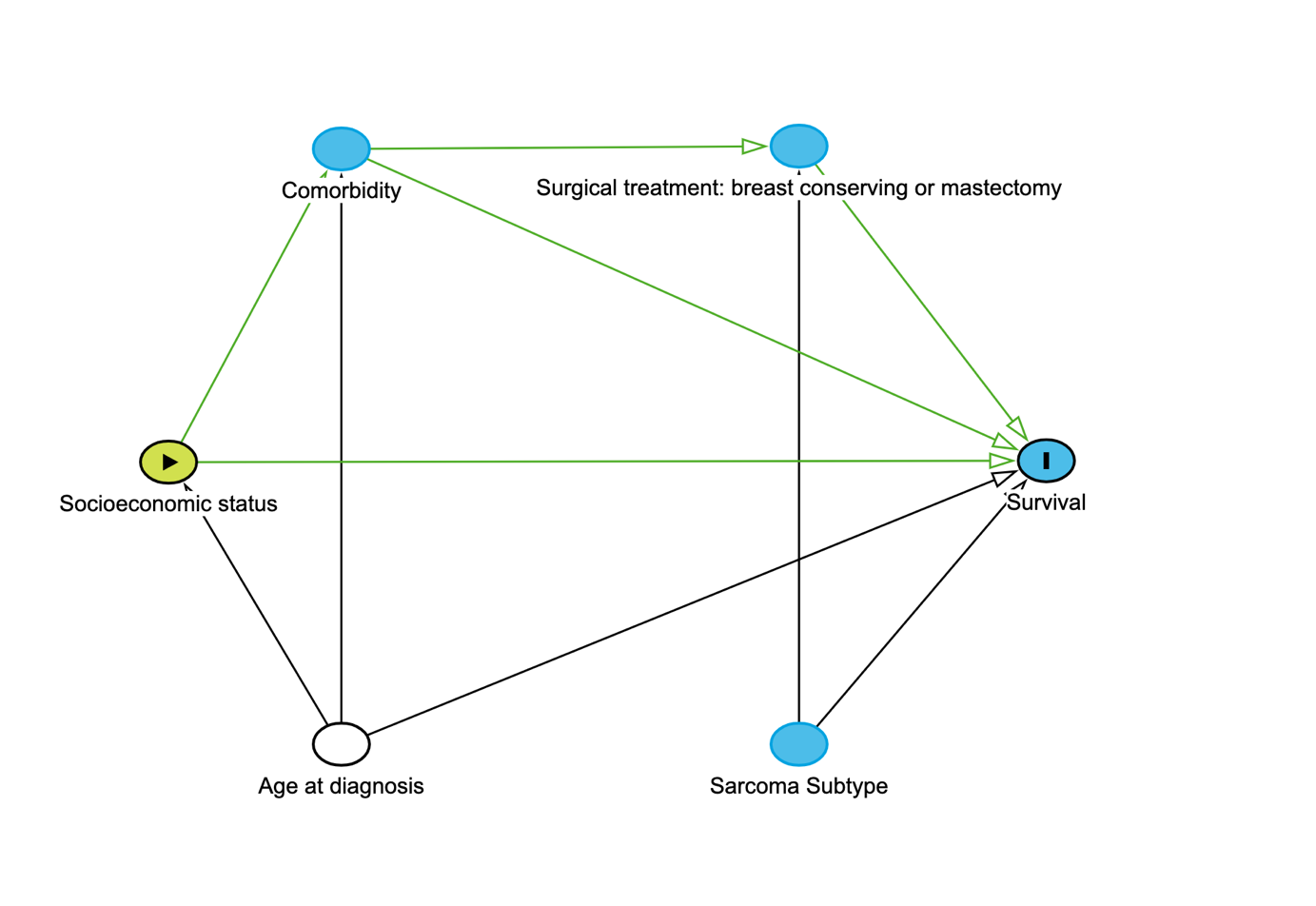


**eFigure 1.** DAG model. Correctly adjusted Directed Acyclic Graph (DAG), displaying possible causal associations to build a statistical model. Exposure: Socioeconomic status; Outcome: Survival. Adjusted: Age at diagnosis. Minimal sufficient adjustment sets containing Age at diagnosis for estimating the total effect of Socioeconomic status on Survival.

**
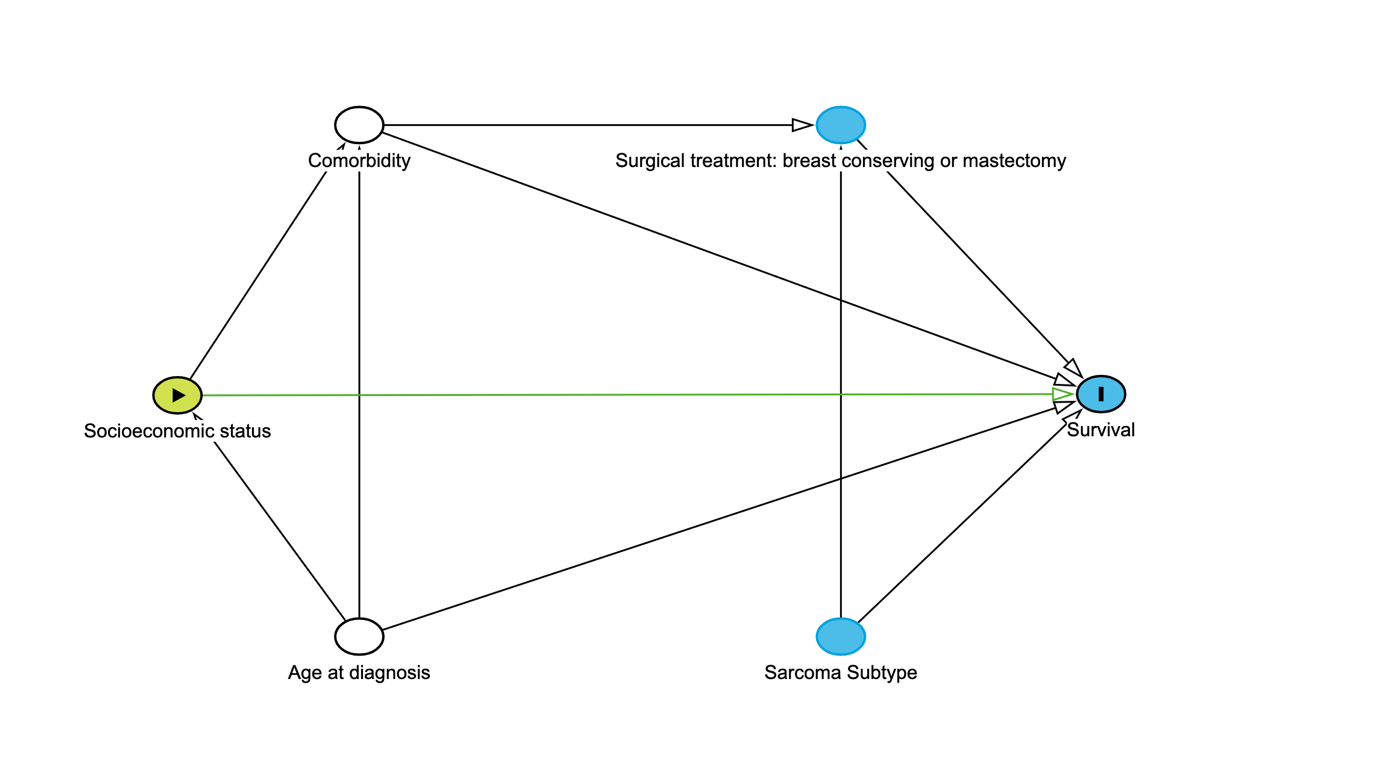
**

**eFigure 2.** Alternate DAG model. Correctly adjusted Directed Acyclic Graph (DAG), displaying possible causal associations to build a statistical model. Exposure: Socioeconomic status; Outcome: Survival. Adjusted: Age at diagnosis and Comorbidity. Minimal sufficient adjustment sets containing Age at diagnosis and Comorbidity for estimating the direct effect of Socioeconomic status on Survival.

**eTable 1. Morphologic diagnosis according to ICD-O/2**

| Morphologic diagnosis |  |  |  |  |  |
| --- | --- | --- | --- | --- | --- |
| according to ICD-O/2 |  |  |  |  |  |
|  |  | Tumor classification | |  |  |
|  | Phyllodes  borderline | Phyllodes  malignant | Angiosarcoma | Soft-tissue sarcoma | Total |
|  |  |  |  |  |  |
| 88003 | 0 | 0 | 0 | 13 | 13 |
| 88013 | 0 | 0 | 0 | 4 | 4 |
| 88023 | 0 | 0 | 0 | 1 | 1 |
| 88103 | 0 | 0 | 0 | 1 | 1 |
| 88211 | 0 | 0 | 0 | 2 | 2 |
| 88503 | 0 | 0 | 0 | 3 | 3 |
| 88903 | 0 | 0 | 0 | 4 | 4 |
| 89303 | 0 | 0 | 0 | 2 | 2 |
| 89803 | 0 | 0 | 0 | 35 | 35 |
| 89823 | 0 | 0 | 0 | 3 | 3 |
| 90200 | 137 | 0 | 0 | 0 | 137 |
| 90201 | 61 | 0 | 0 | 0 | 61 |
| 90202 | 0 | 3 | 0 | 0 | 3 |
| 90203 | 0 | 176 | 0 | 0 | 176 |
| 91203 | 0 | 0 | 23 | 0 | 23 |
| 91703 | 0 | 0 | 2 | 0 | 2 |
| 91803 | 0 | 0 | 0 | 2 | 2 |
| 95403 | 0 | 0 | 0 | 1 | 1 |
|  |  |  |  |  |  |
| Total | 198 | 179 | 25 | 71 | 473 |

**eTable 2. Patient characteristics by tumor category.**

|  | Tumor category^a^ | | | | | |  |
| --- | --- | --- | --- | --- | --- | --- | --- |
|  | Phyllodes borderline | Phyllodes malignant | | Angiosarcoma | Soft-tissue sarcoma | Total |  |
| N | 198 (41.9%) | | 179 (37.8%) | 25 (5.3%) | 71 (15.0%) | 473 (100.0%) | |
| Age at diagnosis | | |  |  |  |  |  |
| 18-29 | 15 (7.6%) | | 15 (8.4%) | 1 (4.0%) | 2 (2.8%) | 33 (7.0%) |  |
| 30-39 | 23 (11.6%) | | 8 (4.5%) | 1 (4.0%) | 7 (9.9%) | 39 (8.2%) |  |
| 40-49 | 61 (30.8%) | | 57 (31.8%) | 4 (16.0%) | 17 (23.9%) | 139 (29.4%) |  |
| 50-59 | 65 (32.8%) | | 55 (30.7%) | 1 (4.0%) | 25 (35.2%) | 146 (30.9%) |  |
| ≥60 | 34 (17.2%) | | 44 (24.6%) | 18 (72.0%) | 20 (28.2%) | 116 (24.5%) |  |
| Charlson Comorbidity Index | | | |  |  |  |  |
| 0 | 186 (93.9%) | | 164 (91.6%) | 15 (60.0%) | 58 (81.7%) | 423 (89.4%) |  |
| ≥1 | 12 (6.1%) | | 15 (8.4%) | 10 (40.0%) | 13 (18.3%) | 50 (10.6%) |  |
| Immigration Status | | |  |  |  |  |  |
| Swedish | 155 (78.3%) | | 139 (77.7%) | 20 (80.0%) | 56 (78.9%) | 370 (78.2%) |  |
| Non-Swedish | 43 (21.7%) | | 40 (22.3%) | 5 (20.0%) | 15 (21.1%) | 103 (21.8%) |  |
| Marital status | | |  |  |  |  |  |
| Unmarried | 57 (29.1%) | | 48 (27.0%) | 3 (12.0%) | 12 (16.9%) | 120 (25.5%) |  |
| Married | 102 (52.0%) | | 104 (58.4%) | 19 (76.0%) | 40 (56.3%) | 265 (56.4%) |  |
| Registered partnership | 1 (0.5%) | | 0 (0.0%) | 0 (0.0%) | 0 (0.0%) | 1 (0.2%) |  |
| Divorced | 32 (16.3%) | | 22 (12.4%) | 2 (8.0%) | 16 (22.5%) | 72 (15.3%) |  |
| Widow | 4 (2.0%) | | 4 (2.2%) | 1 (4.0%) | 3 (4.2%) | 12 (2.6%) |  |
| Highest attained education | | | |  |  |  |  |
| ≤9 years (primary) | 38 (19.2%) | | 42 (23.5%) | 9 (36.0%) | 16 (22.5%) | 105 (22.2%) |  |
| 10-13 years (secondary) | 85 (42.9%) | | 72 (40.2%) | 12 (48.0%) | 30 (42.3%) | 199 (42.1%) |  |
| >13 years (tertiary) | 70 (35.4%) | | 63 (35.2%) | 4 (16.0%) | 24 (33.8%) | 161 (34.0%) |  |
| Missing | 5 (2.5%) | | 2 (1.1%) | 0 (0.0%) | 1 (1.4%) | 8 (1.7%) |  |
| Household disposable income quintiles | | | | |  |  |  |
| Lowest | 33 (16.7%) | | 22 (12.3%) | 5 (20.0%) | 12 (16.9%) | 72 (15.2%) |  |
| Second | 36 (18.2%) | | 31 (17.3%) | 4 (16.0%) | 14 (19.7%) | 85 (18.0%) |  |
| Third | 35 (17.7%) | | 43 (24.0%) | 6 (24.0%) | 12 (16.9%) | 96 (20.3%) |  |
| Fourth | 41 (20.7%) | | 46 (25.7%) | 5 (20.0%) | 17 (23.9%) | 109 (23.0%) |  |
| Highest | 51 (25.8%) | | 36 (20.1%) | 5 (20.0%) | 16 (22.5%) | 108 (22.8%) |  |
| Missing | 2 (1.0%) | | 1 (0.6%) | 0 (0.0%) | 0 (0.0%) | 3 (0.6%) |  |
| T stage |  | |  |  |  |  |  |
| Tx | 170 (85.9%) | | 114 (63.7%) | 13 (52.0%) | 38 (53.5%) | 335 (70.8%) |  |
| T1 | 19 (9.6%) | | 13 (7.3%) | 5 (20.0%) | 10 (14.1%) | 47 (9.9%) |  |
| T2 | 6 (3.0%) | | 30 (16.8%) | 5 (20.0%) | 14 (19.7%) | 55 (11.6%) |  |
| T3 | 3 (1.5%) | | 18 (10.1%) | 0 (0.0%) | 6 (8.5%) | 27 (5.7%) |  |
| T4 | 0 (0.0%) | | 4 (2.2%) | 2 (8.0%) | 3 (4.2%) | 9 (1.9%) |  |
| Lymph Node status | | |  |  |  |  |  |
| Nx | 169 (85.4%) | | 121 (67.6%) | 13 (52.0%) | 39 (54.9%) | 342 (72.3%) |  |
| N0 | 29 (14.6%) | | 55 (30.7%) | 11 (44.0%) | 27 (38.0%) | 122 (25.8%) |  |
| N1 | 0 (0.0%) | | 3 (1.7%) | 1 (4.0%) | 5 (7.0%) | 9 (1.9%) |  |
| Distant metastasis | | |  |  |  |  |  |
| Mx | 166 (83.8%) | | 109 (60.9%) | 13 (52.0%) | 40 (56.3%) | 328 (69.3%) |  |
| M0 | 32 (16.2%) | | 69 (38.5%) | 11 (44.0%) | 29 (40.8%) | 141 (29.8%) |  |
| M1 | 0 (0.0%) | | 1 (0.6%) | 1 (4.0%) | 2 (2.8%) | 4 (0.8%) |  |
| Surgical treatment | | |  |  |  |  |  |
| Breast conserving surgery | 177 (89.4%) | | 91 (50.8%) | 9 (36.0%) | 28 (39.4%) | 305 (64.5%) |  |
| Mastectomy | 21 (10.6%) | | 88 (49.2%) | 16 (64.0%) | 43 (60.6%) | 168 (35.5%) |  |
| Time period | | |  |  |  |  |  |
| 1993-1999 | 48 (24.2%) | | 47 (26.3%) | 2 (8.0%) | 13 (18.3%) | 110 (23.3%) |  |
| 2000-2009 | 80 (40.4%) | | 54 (30.2%) | 10 (40.0%) | 33 (46.5%) | 177 (37.4%) |  |
| 2010-2018 | 70 (35.4%) | | 78 (43.6%) | 13 (52.0%) | 25 (35.2%) | 186 (39.3%) |  |
|  |  | |  |  |  |  |  |

^a^ All percentages represent column proportions within each tumor category subgroup.
Data source: Swedish Cancer Register, Cause of Death Register, National Patient Register, and LISA database. Study population: Women aged 18–70 years diagnosed with an incident sarcoma of the breast in Sweden, 1993–2018, with follow-up through December 31, 2019.

**eTable 3. Patient characteristics by income level.**

|  | Household disposable income quintiles^a^ | | | | | | |
| --- | --- | --- | --- | --- | --- | --- | --- |
|  | First | Second | Third | Fourth | Fifth | Missing | Total |
| N (%) | 72 (15.2) | 85 (18.0) | 96 (20.3) | 109 (23.0) | 108 (22.8) | 3 (0.6) | 473 (100.0) |
| Age at diagnosis | |  |  |  |  |  |  |
| 18-29 | 11 (15.3) | 7 (8.2) | 5 (5.2) | 4 (3.7) | 5 (4.6) | 1 (33.3) | 33 (7.0) |
| 30-39 | 5 (6.9) | 7 (8.2) | 9 (9.4) | 8 (7.3) | 10 (9.3) | 0 (0.0) | 39 (8.2) |
| 40-49 | 15 (20.8) | 23 (27.1) | 22 (22.9) | 33 (30.3) | 45 (41.7) | 1 (33.3) | 139 (29.4) |
| 50-59 | 18 (25.0) | 25 (29.4) | 28 (29.2) | 40 (36.7) | 34 (31.5) | 1 (33.3) | 146 (30.9) |
| ≥60 | 23 (31.9) | 23 (27.1) | 32 (33.3) | 24 (22.0) | 14 (13.0) | 0 (0.0) | 116 (24.5) |
| Tumor category | |  |  |  |  |  |  |
| Phyllodes borderline | 33 (45.8) | 36 (42.4) | 35 (36.5) | 41 (37.6) | 51 (47.2) | 2 (66.7) | 198 (41.9) |
| Phyllodes malignant | 22 (30.6) | 31 (36.5) | 43 (44.8) | 46 (42.2) | 36 (33.3) | 1 (33.3) | 179 (37.8) |
| Angiosarcoma | 5 (6.9) | 4 (4.7) | 6 (6.2) | 5 (4.6) | 5 (4.6) | 0 (0.0) | 25 (5.3) |
| Soft-tissue sarcoma | 12 (16.7) | 14 (16.5) | 12 (12.5) | 17 (15.6) | 16 (14.8) | 0 (0.0) | 71 (15.0) |
| Charlson Comorbidity Index | | |  |  |  |  |  |
| 0 | 59 (81.9) | 77 (90.6) | 83 (86.5) | 101 (92.7) | 100 (92.6) | 3 (100.0) | 423 (89.4) |
| ≥1 | 13 (18.1) | 8 (9.4) | 13 (13.5) | 8 (7.3) | 8 (7.4) | 0 (0.0) | 50 (10.6) |
| Immigration Status | |  |  |  |  |  |  |
| Swedish | 46 (63.9) | 71 (83.5) | 73 (76.0) | 88 (80.7) | 91 (84.3) | 1 (33.3) | 370 (78.2) |
| Non-Swedish | 26 (36.1) | 14 (16.5) | 23 (24.0) | 21 (19.3) | 17 (15.7) | 2 (66.7) | 103 (21.8) |
| Marital status | |  |  |  |  |  |  |
| Unmarried | 35 (48.6) | 33 (38.8) | 21 (21.9) | 19 (17.4) | 12 (11.1) | 0 (.) | 120 (25.5) |
| Married | 8 (11.1) | 24 (28.2) | 59 (61.5) | 81 (74.3) | 93 (86.1) | 0 (.) | 265 (56.4) |
| Registered partnership | 0 (0.0) | 0 (0.0) | 0 (0.0) | 0 (0.0) | 1 (0.9) | 0 (.) | 1 (0.2) |
| Divorced | 25 (34.7) | 24 (28.2) | 12 (12.5) | 9 (8.3) | 2 (1.9) | 0 (.) | 72 (15.3) |
| Widow | 4 (5.6) | 4 (4.7) | 4 (4.2) | 0 (0.0) | 0 (0.0) | 0 (.) | 12 (2.6) |
| Highest attained education | | |  |  |  |  |  |
| ≤9 years (primary) | 19 (26.4) | 28 (32.9) | 24 (25.0) | 25 (22.9) | 9 (8.3) | 0 (0.0) | 105 (22.2) |
| 10-13 years (secondary) | 29 (40.3) | 35 (41.2) | 48 (50.0) | 50 (45.9) | 36 (33.3) | 1 (33.3) | 199 (42.1) |
| >13 years (tertiary) | 21 (29.2) | 22 (25.9) | 23 (24.0) | 34 (31.2) | 61 (56.5) | 0 (0.0) | 161 (34.0) |
| Missing | 3 (4.2) | 0 (0.0) | 1 (1.0) | 0 (0.0) | 2 (1.9) | 2 (66.7) | 8 (1.7) |
| T category |  |  |  |  |  |  |  |
| Tx | 49 (68.1) | 49 (57.6) | 71 (74.0) | 82 (75.2) | 82 (75.9) | 2 (66.7) | 335 (70.8) |
| T1 | 10 (13.9) | 11 (12.9) | 7 (7.3) | 9 (8.3) | 9 (8.3) | 1 (33.3) | 47 (9.9) |
| T2 | 6 (8.3) | 14 (16.5) | 12 (12.5) | 13 (11.9) | 10 (9.3) | 0 (0.0) | 55 (11.6) |
| T3 | 4 (5.6) | 8 (9.4) | 5 (5.2) | 4 (3.7) | 6 (5.6) | 0 (0.0) | 27 (5.7) |
| T4 | 3 (4.2) | 3 (3.5) | 1 (1.0) | 1 (0.9) | 1 (0.9) | 0 (0.0) | 9 (1.9) |
| Lymph Node status | |  |  |  |  |  |  |
| Nx | 55 (76.4) | 52 (61.2) | 66 (68.8) | 82 (75.2) | 85 (78.7) | 2 (66.7) | 342 (72.3) |
| N0 | 14 (19.4) | 31 (36.5) | 28 (29.2) | 26 (23.9) | 22 (20.4) | 1 (33.3) | 122 (25.8) |
| N1 | 3 (4.2) | 2 (2.4) | 2 (2.1) | 1 (0.9) | 1 (0.9) | 0 (0.0) | 9 (1.9) |
| Distant metastasis | |  |  |  |  |  |  |
| Mx | 49 (68.1) | 50 (58.8) | 64 (66.7) | 82 (75.2) | 81 (75.0) | 2 (66.7) | 328 (69.3) |
| M0 | 21 (29.2) | 35 (41.2) | 31 (32.3) | 27 (24.8) | 26 (24.1) | 1 (33.3) | 141 (29.8) |
| M1 | 2 (2.8) | 0 (0.0) | 1 (1.0) | 0 (0.0) | 1 (0.9) | 0 (0.0) | 4 (0.8) |
| Surgical treatment | |  |  |  |  |  |  |
| Breast conserving surgery | 48 (66.7) | 55 (64.7) | 58 (60.4) | 66 (60.6) | 75 (69.4) | 3 (100.0) | 305 (64.5) |
| Mastectomy | 24 (33.3) | 30 (35.3) | 38 (39.6) | 43 (39.4) | 33 (30.6) | 0 (0.0) | 168 (35.5) |
| Time period | |  |  |  |  |  |  |
| 1993-1999 | 11 (15.3) | 15 (17.6) | 25 (26.0) | 31 (28.4) | 28 (25.9) | 0 (0.0) | 110 (23.3) |
| 2000-2009 | 33 (45.8) | 36 (42.4) | 31 (32.3) | 34 (31.2) | 42 (38.9) | 1 (33.3) | 177 (37.4) |
| 2010-2018 | 28 (38.9) | 34 (40.0) | 40 (41.7) | 44 (40.4) | 38 (35.2) | 2 (66.7) | 186 (39.3) |

^a^All percentages represent column proportions within each income level subgroup.

Data source: Swedish Cancer Register, Cause of Death Register, National Patient Register, and LISA database. Study population: Women aged 18–70 years diagnosed with an incident sarcoma of the breast in Sweden, 1993–2018, with follow-up through December 31, 2019.

**eFigure 3. Kaplan-Meier survival estimates by income quintiles for different tumor categories.**

Data source: Swedish Cancer Register, Cause of Death Register, National Patient Register, and LISA database. Study population: Women aged 18–70 years diagnosed with an incident sarcoma of the breast in Sweden, 1993–2018, with follow-up through December 31, 2019.
